# Documented *β*-Lactam Allergy and Risk for Cesarean Surgical Site Infection

**DOI:** 10.1155/2022/5313948

**Published:** 2022-03-02

**Authors:** Courtney Johnston, Amy Godecker, Daniel Shirley, Kathleen M. Antony

**Affiliations:** ^1^University of Wisconsin School of Medicine and Public Health, 750 Highland Ave, Madison, WI 53726, USA; ^2^Department of Obstetrics and Gynecology, University of Wisconsin School of Medicine and Public Health, 1010 Mound Street Madison, WI 53715, USA; ^3^Division of Infectious Diseases, Department of Medicine, University of Wisconsin School of Medicine and Public Health, 1685 Highland Avenue, 5158 Medical Foundation Centennial Building, Madison WI 53705-2281, USA

## Abstract

**Objective:**

To examine the relationship between documented *β*-lactam allergy and cesarean delivery (CD) surgical site infection (SSI). *Study Design*. We conducted a retrospective cohort analysis of women who underwent CD at Ben Taub Hospital and Texas Children's Pavilion for Women (Houston, TX) from August 1, 2011, to December 31, 2019. The primary exposure was a documented *β*-lactam allergy, and the second exposure of interest was the type of perioperative antibiotic received. The primary outcome was the prevalence of SSI. Maternal characteristics were stratified by the presence or absence of a documented *β*-lactam allergy, and significance was evaluated using Pearson's chi-squared test for categorical variables and *t*-test for continuous variables. A logistic regression model estimated odds of SSI after adjusting for possible confounders.

**Results:**

Of the 12,954 women included, 929 (7.2%) had a documented *β*-lactam allergy while 12,025 (92.8%) did not. Among the 929 women with a *β*-lactam allergy, 495 (53.3%) received non-*β*-lactam perioperative prophylaxis. SSI occurred in 38 (4.1%) of women who had a *β*-lactam allergy versus 238 (2.0%) who did not (*p* ≤ 0.001). *β*-Lactam allergy was associated with higher odds of SSI compared to no allergy (adjusted odds ratio (aOR) = 1.97; 95%confidence interval (CI) = 1.24-3.14; *p* = 0.004) after controlling for age, race, ethnicity, insurance status, delivery body mass index (BMI), tobacco use, intra-amniotic infection in labor, duration of membrane rupture, preterm delivery, delivery indication, diabetes, hypertension, group B Streptococcus colonization, and type of perioperative antibiotic received.

**Conclusion:**

The presence of a *β*-lactam allergy is associated with increased odds of developing a CD SSI after controlling for possible confounders, including the type of perioperative antibiotic received.

## 1. Introduction

Penicillin allergy is the most common drug allergy in the United States, with a 10-20% reported prevalence in hospitalized patients [1–6]. However, recent literature has shown that approximately 88-99% of these patients do not have an immediate hypersensitivity reaction [1–5]. Penicillin is one type of beta-lactam antibiotic. Preoperative antibiotic prophylaxis remains one of the most important strategies for preventing postoperative infection or surgical site infection (SSI) [3, 7]. The American College of Obstetricians and Gynecologists (ACOG) recommends *β*-lactam containing regimens over non-*β*-lactam regimens. Antimicrobial agents used for surgical prophylaxis ideally prevent SSI, prevent SSI-related morbidity and mortality, reduce the duration and cost of healthcare, produce no adverse effects, and have no adverse consequences on the microbial flora of the patient or hospital [8]. To achieve these goals, the agent should be active against the pathogens most likely to contaminate the surgical site, be administered at an adequate dosage and timed to ensure adequate serum and tissue concentrations during the time of contamination, be safe, and be used for the shortest effective period to minimize adverse effects and the development of resistance [8]. Specifically, due to its favorable cost, antibacterial activity, safety, and pharmacokinetic profile, cefazolin is the optimal choice of antibiotic prophylaxis [8, 9]. The cross-reactivity of penicillin with cephalosporins is generally low as well, making it a safe option [10]. However, if a significant penicillin allergy is present (anaphylaxis, angioedema, respiratory distress, or urticaria), a second-line therapy of clindamycin and gentamicin is recommended [11]. These agents are typically less effective, leaving these patients at higher risk for SSI [3, 11].

SSI is defined by the Centers for Disease Control and Prevention (CDC) as an infection that occurs within 30 days of a surgical procedure [12]. SSIs are potentially preventable complications that are associated with excess morbidity and mortality [2, 7, 12–16]. SSIs constitute the largest portion of hospital-associated infection- (HAI-) related costs nationally and are associated with significant economic burden in terms of an extended length of stay, hospital readmissions, and increased treatment cost [2, 7, 12–16].

The effects of penicillin allergy on SSI have been studied in many surgical procedures, including hip and knee arthroplasty, hysterectomy, colon surgery, and coronary artery bypass grafting; however, minimal research has been done regarding obstetric populations [3, 17, 18]. Cesarean delivery (CD) is the most common major surgery in the United States, with over one-third of babies in the US delivered via CD, and these procedures carry a high risk of SSI, approximately 2-19% of CD [1–3, 7, 19–22].

The purpose of this study is to examine the association between the presence of a documented *β*-lactam allergy and the prevalence of SSI following CD. We hypothesize that patients with a documented *β*-lactam allergy will have a higher odds of developing SSI than those who do not have a *β*-lactam allergy.

## 2. Materials and Methods

### 2.1. Data Source

This study was reviewed by the Institutional Review Board at the University of Wisconsin and deemed to be exempt (submission ID 2020-0535). We performed a retrospective cohort study of women who underwent CD from August 1, 2011, to December 31, 2019, at Ben Taub Hospital and Texas Children's Pavilion for Women (Harris Health System, Houston, TX) utilizing deidentified data of participants enrolled in PeriBank. PeriBank is a perinatal database and biospecimen repository housed at Baylor College of Medicine (BCM) and funded by the generous support of the Departments of Obstetrics and Gynecology and Pathology and Laboratory Medicine at Texas Children's Hospital and Baylor College of Medicine (BCM) via the PeriBank protocol (BCM IRB H-26364, Dr. Kjersti Aagaard, PI). A full description of this biobank's recruitment protocols, review processes, and available data and specimens is available elsewhere and is also described in Acknowledgements [23]. The institution that houses the biobank has SSI protocols and guidelines in place which reflect the guidance of ACOG [24].

### 2.2. Subjects

Subjects were recruited by trained PeriBank study personnel who approached eligible gravidae at the time of admission for delivery. After obtaining consent, clinical information is manually extracted from the electronic medical record and accompanying prenatal records. Data entry into PeriBank is completed by trained study nurses and research staff and is completed at the time of delivery; the chart is later updated to ensure the capture of relevant information for six weeks postpartum. A subset of charts is routinely audited by a board-certified maternal-fetal medicine physician scientist at BCM to ensure quality of the data. However, due to the volume of births at these two institutions, review of all charts is not feasible; only a subset is reviewed. We extracted and analyzed clinical data for this study, including maternal characteristics, demographics, behaviors, and obstetric and neonatal outcomes from PeriBank.

### 2.3. Exposure

The primary exposure was the presence of a *β*-lactam allergy, defined as a documented allergy to a penicillin or penicillin derivative, cephalosporin, or carbapenem at the time of CD. The PeriBank dataset included only the documented allergy not the specific type of reaction; thus, the type and significance of the allergy were not known. Therefore, many with a documented allergy were appropriate to receive beta-lactam antibiotics and were noted to have received these.

The secondary exposure of interest was the type of perioperative antibiotic received. This was classified as either a *β*-lactam (penicillin, cephalosporin, or carbapenem) or a non-*β*-lactam. Per institutional protocol at BCM and consistent with national guidelines, women with significant penicillin or cephalosporin allergies, such as anaphylaxis, angioedema, respiratory distress, or urticaria, received non-*β*-lactam antibiotics such as clindamycin plus aminoglycoside [7]. Women with less significant penicillin allergies generally receive a cephalosporin per institutional protocol and following national guidelines [11, 24]. The institutional protocol was also consistent with national guidelines to administer antibiotics within 60 minutes of CD [11, 24]; however, the timing of administration was not entered into PeriBank. Finally, during the period of this study, use of azithromycin for cesarean births during labor or after membrane rupture became routine and was incorporated into this institution's standard protocol in 2016 [25].

### 2.4. Study Outcomes

The primary outcome was the 30-day incidence of SSI, as defined by the National Healthcare Safety Network (NHSN) for CD, including all SSI types: superficial, deep, and organ space [12]. However, SSI is not a discrete field in the PeriBank dataset. Therefore, SSI was determined through manual review of PeriBank for postpartum and readmission infections and was defined as a composite of endometritis, cellulitis, pelvic inflammatory disease (as this likely represented endometritis), sepsis, abdominal and pelvic abscesses, and wound infection. Endometritis prior to discharge was defined as a uterine infection that occurred during the delivery hospitalization and not as a surgical site infection due to potential miscoding of intra-amniotic infection. Postpartum endometritis, diagnosed after the index delivery hospital discharge, was included as a component of the primary outcome.

### 2.5. Data Synthesis (Covariates and Confounders)

Race, ethnicity, gravidity, parity, BMI, tobacco use, insurance status, medical comorbidities (hypertension, cardiovascular disease, and diabetes), maternal infections, and colonization (group B Streptococcus colonization, intra-amniotic infection, endometritis, and sexually transmitted infections) were identified through the chart review. Those with group B Streptococcus colonization were treated with intravenous penicillin or an appropriate alternative depending upon allergies and sensitivities if they experienced labor [26]. These patients subsequently received perioperative antibiotics per hospital guidelines once the decision was made to proceed with cesarean and within 60 minutes before starting the surgery. Chronic and gestational hypertension was recorded separately. Hypertensive disorders of pregnancy were diagnosed based upon the contemporary diagnostic criteria which varied over the 9 years of data collected [27–29]. Pregestational diabetes included patients with a history of type I or type II diabetes; both pregestational diabetes and gestational diabetes were diagnosed based upon the contemporary diagnostic criteria for the period [30–33].

Duration of membrane rupture was calculated by subtracting the date and time of rupture of membranes from the date and time of delivery. Delivery indications were defined as follows: scheduled cesarean with a previous CD; scheduled—other (e.g., breech and previa); unscheduled—fetal related (e.g., nonreassuring fetal status); unscheduled—labor related; and other. Whether the delivery occurred during labor was also noted. Both the indications for cesarean and occurrence of labor were identified by chart review by trained PeriBank personnel.

A manual review of allergies entered into the PeriBank database by PeriBank staff was performed by one coauthor of this study (CJ) for each participant. It was recorded as a *β*-lactam allergy if the documented allergy was to penicillin, cephalosporin, or carbapenem. The type of perioperative antibiotic received was also entered into PeriBank by PeriBank staff following manual review of the home institution's electronic health record. For this study, the antibiotics received as listed in PeriBank were also reviewed by one coauthor (CJ). However, this study team did not have any direct access to the home institution's electronic health record, only the PeriBank dataset. Penicillins, cephalosporins, and carbapenems were considered a *β*-lactam.

### 2.6. Data Analysis

PeriBank is organized as one record per baby delivered. To adjust for women who were in the dataset for more than one pregnancy or multiple infants per pregnancy, we randomly selected one record per woman. We stratified maternal characteristics by the presence or absence of a documented *β*-lactam allergy and assessed significant associations using Pearson's chi-squared test for independence for categorical variables and Student's *t*-test for continuous variables with normal distribution and Wilcoxon rank-sum test for those without normal distribution. We used multivariable logistic regression to determine the association between a reported *β*-lactam allergy and SSI. In the obstetrical and gynecological literature, covariates were selected based on known risk factors associated with a reported *β*-lactam allergy and SSI [15, 18–20, 34, 35]. These covariates included age, race, ethnicity, delivery BMI, tobacco use, intra-amniotic infection, duration of membrane rupture, preterm delivery, delivery indication, pregestational and gestational diabetes, hypertension, and type of perioperative antibiotic received. Group B Streptococcus colonization and insurance status were included due to either statistically significant differences in baseline characteristics or concerns that these may be associated with SSI based upon literature review [15, 18–20, 34, 35]. Given the number of covariates, we conducted the Pearson chi-squared goodness-of-fit test and the Hosmer-Lemeshow goodness-of-fit test. All statistical analyses were performed using Stata/SE 16.1 statistical software, and a 2-sided *p* value of <0.05 was considered statistically significant. The dataset for this project is available upon reasonable request from the corresponding author; access to the PeriBank dataset is available separately [23].

## 3. Results

### 3.1. Maternal Characteristics

There were 16,831 records for babies delivered by cesarean during the study period, and 3,877 were excluded after randomly selecting one baby per woman. Of the 12,954 women included, 929 (7.2%) had a reported *β*-lactam allergy, while 12,025 (92.8%) did not. Among women with a reported *β*-lactam allergy, 495 (53.3%) received non-*β*-lactam prophylaxis compared to 1.1% of women without a reported *β*-lactam allergy (*p* < 0.001). Women with a documented *β*-lactam allergy were older (31.4 vs. 30.5 years, *p* < 0.001) and had significant racial (*p* = 0.017) and ethnic (*p* ≤ 001) differences compared to women without a documented allergy. Participants with a *β*-lactam allergy were more likely to have private insurance (58.1% vs. 34.4%, *p* < 0.001), while those without a *β*-lactam allergy were more likely to have CHIP/Medicaid (61.0% vs. 39.9%, *p* < 0.001). Indications for CD were also significantly associated with documented *β*-lactam allergy (*p* = 0.046). (Table 1).

Maternal characteristics, including delivery BMI, cesarean after the onset of labor, hypertension, pregestational diabetes, and tobacco use, were similar for women with and without a documented *β*-lactam allergy. All obstetric characteristics, including gestational age, preterm birth, cesarean hysterectomy, and postoperative length of stay, were also similar for both groups of women. Finally, group B Streptococcus colonization, intra-amniotic infection, and sexually transmitted infections (STI) were similar between the two groups.

### 3.2. Surgical Site Infection

SSI occurred in 38 (4.1%) of women with a documented *β*-lactam allergy versus 236 (2.0%) without a *β*-lactam allergy (*p* ≤ 0.001) (Table 2). Of the subtypes of SSI, endometritis, wound infections, and abscesses occurred in a higher percentage of women with *β*-lactam allergy. In the multivariable logistic regression model controlling for the aforementioned covariates, *β*-lactam allergy was associated with increased odds of SSI (adjusted odds ratio (aOR) = 1.97; 95%confidence interval (CI) = 1.24-3.14; *p* = 0.004) (Table 3). Perioperative non-*β*-lactam prophylaxis was also associated with increased odds of SSI (aOR = 1.43; 95%CI = 1.01–2.02; *p* = 0.045). Other important variables significantly associated with increased odds of SSI included intra-amniotic infection, preterm delivery, and hypertension. Group B Streptococcus colonization was associated with a decreased odds of SSI (aOR = 0.69; 95%CI = 0.49–0.98; *p* = 0.039). BMI was associated with a slightly increased odds of SSI (aOR = 1.02; 95%CI = 1.00-1.04; *p* = 0.014). Age, race, ethnicity, insurance status, duration of membrane rupture, maternal tobacco use, and diabetes were not significantly associated with SSI.

A mediator analysis suggests that the association between the documented beta-lactam and SSI is unaffected by the receipt of non-beta-lactam antibiotics. The coefficient and *p* value for a model without inclusion of non-beta-lactam in the model is *β* = 0.956 and *p* = .792, respectively, while the coefficient and *p* value for the model including non-beta-lactam is *β* = 0.952 and *p* = 0.778, respectively, for a mediated or indirect effect close to 0. Given the number of covariates, we conducted the Pearson chi-squared goodness-of-fit test with a resulting Prob > chi^2^ = 0.6864. It is not significant, so we do not reject our model. However, because the number of covariate patterns is close to the number of observations (because of the linear terms included like age and BMI), Pearson might not be appropriate. We also conducted the Hosmer-Lemeshow GOF test with 10 groups. For this test, Prob > chi^2^ = 0.2168; the test is not significant, so we cannot reject our model.

## 4. Discussion

This large retrospective cohort study showed that a documented *β*-lactam allergy was associated with increased odds of CD SSI after controlling for numerous important confounders including the type of perioperative antibiotic received. Participants with a reported *β*-lactam allergy were more likely to receive a second-line antibiotic regimen compared to their nonallergic counterparts, and the use of non-*β*-lactam prophylaxis was associated with a statistically significant increased odds of CD SSI.

One unexpected finding was that the relationship between perioperative *β*-lactam and SSI was not statistically significant. This may be related to the fact that there were 2,127 (16.4%) women who reportedly received no antibiotic prophylaxis. This most likely reflects a documentation error in the database wherein our results have underestimated the number of participants who received *β*-lactam antibiotic prophylaxis. Participants with an allergy were also more likely to have private insurance than their nonallergic counterparts; private insurance (and factors that covary with private insurance such as provider mix) is associated with a lower surgical site infection rate [36]. One possible explanation is access to care, where those with private insurance may be more inclined to seek care and receive an antibiotic in the first place as well as subsequent care and documentation if they were to develop an allergy. Group B Streptococcus colonization was associated with decreased odds of SSI, consistent with the findings of other authors in the setting of routine prophylaxis [37]. Women with group B Streptococcus most likely received additional antibiotic therapy, particularly if they labored prior to their cesarean birth. The smoking rate during pregnancy in this population (2.0%) was also well below the national average (7.1%) [38]. Smoking has been associated with increased risk of SSI in many surgical procedures including cesarean delivery [39, 40].

Literature shows that administration of non-*β*-lactam prophylaxis increases the risk of adverse surgical outcomes including SSI [1–3, 18, 41]. Hopkins et al. and Harris et al. separately retrospectively analyzed the prevalence of CD SSI between women who received perioperative *β*-lactam vs. non-*β*-lactam prophylaxis [1, 2]. Both studies showed that patients who received non-*β*-lactam antibiotics were more likely to have a wound complication, including infection, versus those who received *β*-lactam antibiotics (14.6% vs. 6.7% by Hopkins et al. and 15% vs. 7% by Harris et al.) [1, 2]. Thus, these studies identified an increased odd of SSI with non-*β*-lactam prophylaxis consistent with our findings (aOR = 1.43; 95%CI = 1.01–2.02; *p* = 0.045) [1, 2]. However, our study is novel in that it explicitly evaluated the relationships between SSI and *β*-lactam allergy in addition to the type of prophylaxis given.

ACOG recommends a single dose of first-generation cephalosporin for perioperative prophylaxis administered within 60 minutes of the start of CD [42]. For the 10-20% of women with a history of a significant penicillin or cephalosporin allergy, a single-dose of clindamycin with an aminoglycoside is recommended [1–6, 11]. This is despite literature showing that approximately 88-99% of these patients do not actually have an immediate hypersensitivity reaction, and ACOG states that allergy testing may be considered [1–5, 27]. Furthermore, in patients with true penicillin allergies, the cross-reactivity to a cephalosporin is estimated to be between 0.9 and 17% depending on the type of cephalosporin. This cross-reactivity is primarily driven by the similarity between beta-lactam R1 side chains [10, 43, 44]. For example, one meta-analysis showed that cephalosporins that share identical R1 side chains with penicillin had a cross-reactivity of 16.5% versus 2.1% in cephalosporins with a lower R1 side chain similarity score [44]. Similarly, the cross-reactivity risk between penicillins and carbapenems is even lower around 0.8%-1.0% [10, 43–45]. Given the low cross reactivity, some authors have administered cephalosporins to patients with penicillin allergies without skin testing using simple screening tools to screen for significant allergies [46, 47]. While this is a reasonable approach, penicillin skin testing remains a consideration, particularly when the nature of the allergy is unclear.

Being designated as having a *β*-lactam allergy during pregnancy has been associated with a higher risk of complications including postcesarean wound complications and longer length of hospital stay [1, 48]. Given this information and our findings, it may be beneficial for providers to include a comprehensive allergy history, including specific reactions, and consider testing for those with a documented *β*-lactam allergy during prenatal visits, which has been proven to be feasible and safe [11]. Studies have identified a wide degree of variability in the documentation of *β*-lactam allergies suggesting that up to 95% were misclassified [49]. A thorough allergy history has been shown to be effective in removing an inappropriately documented allergy from the electronic health record [45]. Reporting the specific allergic reaction in the electronic health record for patients with a documented *β*-lactam allergy has also been demonstrated to result in more patients being given first-line *β*-lactam antibiotics [49].

Penicillin skin testing improves antibiotic choice for obstetric populations and is safe to perform during pregnancy [50–52]. A negative penicillin skin test carries a high predictive value (>95% that approaches 100%) when combined with an oral amoxicillin challenge [50]. This testing is also cost-effective. Patients who received a skin test and were switched to a *β*-lactam had an overall cost savings of approximately $297 per person solely based on the savings from switching antibiotics [53]. On a larger scale, penicillin skin testing has been shown to significantly reduce annual healthcare expenditures [45]. A recent meta-analysis showed that patients with a reported penicillin allergy had higher inpatient hospitalizations costing on average $1,145-$4,254 more per patient [54].

Strengths of this paper include the use of a large, validated database of women who underwent CDs. The institutions represented in this dataset cover women across the socioeconomic spectrum and reflect the racial and ethnic composition of the fourth largest city in the United States; thus, they may be generalizable to other large urban settings. Limitations of this study include the fact that specific allergic reactions were not documented; therefore, we were unable to further classify allergies as high or low risk. However, we were able to manually validate key variables, including an allergy, SSI, and type of perioperative antibiotic received. Data on antibiotic administration in the database was absent for 2,127 women; it is not clear whether this reflects a data abstraction error (as data entry into PeriBank is performed manually) or whether these women indeed did not receive preoperative antibiotics [23]. The dataset also lacked information on the dosage and timing of perioperative antibiotic prophylaxis, so we were unable to verify that ACOG recommendations were being followed, which could impact our primary outcome. However, the hospital did have established protocols that align with ACOG's recommendations [11, 24]. Given that SSI only occurred in 274 participants, we could not simultaneously account for all possible confounders in the relationship between SSI and *β*-lactam allergy. Together with the missing data on antibiotic administration, the prevalence of *β*-lactam allergy in this population is lower than that reported elsewhere, and the smoking rate is low, all of which suggest the potential for missing data in the PeriBank dataset [18, 52]. Finally, this database includes two institutions and there may be unaccounted for institutional differences that impact antibiotic administration or reliable recording of allergies or medication administration. However, both institutions are affiliated with the same academic institution, use the same electronic health record system, and follow the same practice guidelines. Both institutions also have the same practitioners (the same resident physicians and attending physicians), all of which should limit any potential differences.

Nevertheless, we were able to adjust for key confounders identified in the previous literature. This dataset was also limited in the descriptions of the documented infections; therefore, it is possible that we misestimated the number of participants with an SSI. Finally, this data represents the patients and practices of Ben Taub General Hospital and Texas Children's Pavilion for Women (Harris Health System, Houston, TX). These two institutions reflect a single academic affiliation which may limit the applicability of these findings to other settings. It also ensures a more uniform surgical practice and standardized perioperative antibiotic use.

## 5. Conclusions

Women with a documented *β*-lactam allergy had 1.97 increased odds of developing an SSI after CD even after controlling for the type of perioperative antibiotic received. Furthermore, women who received non-*β*-lactam prophylaxis had 1.43 increased odds of developing SSI. There has been limited research evaluating the relationship between *β*-lactam allergy and SSI, even less so in obstetric populations [3, 18]. CD is one of the most common surgical procedures in the United States and carries a high risk of SSI [1, 2, 7, 20–22]. Our findings confirm that beta-lactam allergy is a risk factor for SSI and suggest the need for educational initiatives regarding appropriate preventative measures such as comprehensive allergy history and the need for increased utilization of penicillin skin testing prior to surgery to improve both antibiotic choices and mitigate the risk of adverse health outcomes.

## Figures and Tables

**Table 1:** Maternal demographics and obstetric characteristics for women with and without *β*-lactam allergy.

Characteristic	No *β*-lactam allergy	*β*-lactam allergy	*p* value
	*N* = 12,025 (92.83)	*N* = 929 (7.17)	
Age (mean, SD)	30.5 ± 6.1	31.4 ± 5.6	**<0.001**
Gravidity (mean, SD)	2.64 ± 1.36	2.57 ± 1.36	0.084
Nulliparous (*n*, %)	3,983 (33.12)	352 (37.89)	**0.003**
Prepregnancy BMI (mean, SD)	28.31 ± 7.1	28.13 ± 7.7	0.507
Delivery BMI (mean, SD)	33.59 ± 6.9	33.65 ± 7.1	0.794
Race (*n*, %)			**0.017**
White	8,700 (77.35)	716 (77.07)	
African American	2,422 (20.14)	156 (16.79)	
Asian	596 (4.96)	38 (4.09)	
Other	244 (2.03)	12 (1.29)	
Multiracial	63 (0.52)	7 (0.75)	
Hispanic ethnicity (*n*, %)	6,361 (52.92)	296 (31.86)	**<0.001**
Insurance (*n*, %)			**<0.001**
Public	7,206 (60.95)	365 (39.93)	
Private	4,062 (34.36)	531 (58.10)	
None/other/unknown	555 (4.69)	18 (1.97)	
Cesarean indication (*n*, %)			**0.046**
Scheduled			
Repeat CD	4,665 (38.91)	358 (38.70)	
Other (breech, previa, etc.)	1,574 (13.13)	134 (14.49)	
Unscheduled			
Labor-related	865 (7.22)	78 (8.43)	
Fetal well-being-related	2,333 (19.46)	146 (15.78)	
Other	2,551 (21.28)	209 (22.59)	
Duration of membrane rupture, median [IQR]	1 [0-528]	1 [0-543]	0.391
Cesarean in labor (*n*, %)	3,198 (26.68)	224 (24.22)	0.238
Medical and obstetric comorbidities (*n*, %)			
Hypertension	2,769 (23.0)	229 (24.65)	0.258
Diabetes			
Pregestational diabetes	540 (4.51)	37 (4.00)	0.467
Gestational diabetes	1,378 (11.51)	82 (8.84)	**0.013**
Smoking during pregnancy	239 (1.99)	25 (2.69)	0.144
Obstetric characteristics			
GA at delivery (mean, SD)	38.23 ± 2.66	38.08 ± 2.56	0.106
Preterm birth (*n*, %)	2,075 (17.26)	154 (16.58)	0.597
Cesarean hysterectomy performed (*n*, %)	37 (0.31)	4 (0.43)	0.521
Length of stay (postoperatively) (days, mean, SD)	3.64 ± 3.12	3.75 ± 1.77	0.316
Infections or colonizations (*n*, %)			
Group B Streptococcus colonization	2,697 (22.43)	233 (25.08)	0.063
Infections (any)			
Chorioamnionitis	765 (6.36)	47 (5.06)	0.115
Endometritis prior to discharge	3 (0.02)	0 (0.00)	0.630
Sexually transmitted disease (ex: Chlamydia and gonorrhea)	419 (3.48)	22 (2.37)	0.071
Antibiotics received (*n*, %)			**<0.001**
At least one *β*-lactam antibiotics (includes penicillins and cephalosporins)	9,862 (82.01)	334 (35.95)	
Non-*β*-lactam antibiotic	137 (1.14)	495 (53.28)	
No antibiotics documented in the database	2,026 (16.85)	100 (10.76)	

Bold indicates statistical significance. Student's *t*-test, Wilcoxon rank sum test, and chi-square test were used as appropriate.

**Table 2:** Surgical site infection characteristics.

Characteristic	No *β*-lactam allergy	*β*-lactam allergy	*p* value
	*N* = 12,025 (92.83)	*N* = 929 (7.17)	
Surgical site infection^∗^^,†^	236 (2.0)	38 (4.1)	<0.001
Endometritis	164 (1.4)	25 (2.7)	0.001
Cellulitis^‡^	13 (0.1)	2 (0.2)	0.293
Sepsis^‡^	14 (0.1)	3 (0.3)	0.118
Abscess^‡^	6 (0.05)	3 (0.3)	<0.022
Wound infection	54 (0.4)	12 (1.3)	0.001

^∗^0 had pelvic inflammatory disease listed as the indication for readmission. ^†^Some women had more than one subtype of surgical site infection. ^‡^Fisher's exact test (other results are chi-square test).

**Table 3:** Unadjusted and adjusted association between maternal and obstetric characteristics, including *β*-lactam allergy and surgical site infection.

Covariate	OR for SSI	*p* value	aOR for SSI	*p* value
*β*-Lactam allergy	2.13 (1.50-3.02)	<0.001	1.97 (1.24–3.14)	0.004
Age	0.97 (0.95-0.99)	0.004	0.98 (0.96–1.00)	0.104
Race				
White	Ref^∗^		Ref^∗^	
African American	1.36 (1.02-1.80)	0.033	1.26 (0.82–1.93)	0.290
Asian	1.05 (0.59-1.85)	0.865	1.49 (0.79–2.82)	0.217
Other	1.20 (0.53-2.74)	0.678	1.80 (0.77–4.22)	0.173
Multiracial	2.25 (0.70-7.21)	0.174	2.40 (0.69–8.37)	0.170
Ethnicity				
Non-Hispanic	Ref^∗^		Ref^∗^	
Hispanic	1.00 (0.79–1.27)	0.987	0.98 (0.66–1.47)	0.934
Insurance				
Private	Ref^∗^		Ref^∗^	
Public	1.32 (1.02–1.72)	0.036	1.25 (0.87–1.79)	0.217
None/other/unknown	0.76 (0.37–1.58)	0.462	0.78 (0.34–1.77)	0.559
Delivery BMI	1.04 (1.02-1.05)	<0.001	1.02 (1.00–1.04)	0.014
Smoking during pregnancy	0.71 (0.26–1.91)	0.496	0.48 (0.15–1.545)	0.221
Chorioamnionitis	2.84 (2.03-3.98)	<0.001	1.52 (0.98–2.35)	0.060
Duration of membrane rupture (min)	1.00 (0.99–1.00)	0.739	1.00 (0.99–1.00)	0.736
Preterm birth	1.67 (1.27–2.20)	<0.001	1.47 (1.06–2.05)	0.021
Cesarean indication				
Scheduled	Ref^∗^		Ref^∗^	
Unscheduled—fetal well-being related	1.89 (1.36-2.64)	<0.001	1.65 (1.13–2.41)	0.010
Unscheduled—labor related	4.64 (3.28-6.56)	<0.001	3.60 (2.39–5.42)	<0.001
Other	2.04 (1.49–2.80)	<0.001	1.93 (1.35–2.77)	<0.001
Pregestational diabetes	1.43 (0.87–2.35)	0.158	1.18 (0.68–2.03)	0.557
Gestational diabetes	1.15 (0.80–1.65)	0.441	1.01 (0.67–1.52)	0.960
Hypertension	2.17 (1.69–2.77)	<0.001	1.63 (1.21–2.18)	0.001
Group B Streptococcus colonization	0.70 (0.51–0.96)	0.030	0.69 (0.49–0.98)	0.039
*β*-Lactam received	0.77 (0.58–1.01)	0.060	0.95 (0.68–1.33)	0.778
Non-*β*-lactam received	2.46 (1.90–3.18)	<0.001	1.43 (1.01–2.02)	0.045

## Data Availability

The dataset for this project is available upon reasonable request from the corresponding author.
